# The Association between Nutritional Markers and Heart Rate Variability Indices in Patients Undergoing Chronic Hemodialysis

**DOI:** 10.3390/jcm8101700

**Published:** 2019-10-16

**Authors:** Eric Chien-Hwa Wu, Ya-Ting Huang, Yu-Ming Chang, I-Ling Chen, Chuan-Lan Yang, Show-Chin Leu, Hung-Li Su, Jsun-Liang Kao, Shih-Ching Tsai, Rong-Na Jhen, Chih-Chung Shiao

**Affiliations:** 1 Division of Nephrology, Department of Internal Medicine, Saint Mary’s Hospital Luodong, No. 160, Zhongheng S. Rd., Luodong, Yilan 26546, Taiwanynk123.tw@yahoo.com.tw (Y.-M.C.);; 2 Department of Nursing, Saint Mary’s Hospital Luodong, No. 160, Zhongheng S. Rd., Luodong, Yilan 26546, Taiwan; 3 Saint Mary’s Junior College of Medicine, Nursing and Management, No.100, Ln. 265, Sec. 2, Sanxing Rd., Sanxing Township, Yilan County 266, Taiwan

**Keywords:** autonomic nervous system, chronic kidney disease, heart rate variability, hemodialysis, nutritional markers, PEW syndrome

## Abstract

The associations between nutritional markers and heart rate variability (HRV) are poorly addressed. This study aimed to evaluate whether malnutrition is associated with the altered autonomic nervous system (ANS) function. This cross-sectional study was conducted enrolling 175 patients (100 women, mean age 65.1 ± 12.9 years) receiving chronic hemodialysis in a teaching hospital from June to August 2010. We performed HRV measurements before and during the index hemodialysis and compared these HRV values between two groups categorized by the individual nutritional marker. By using the multivariate generalized estimating equation with adjustment, we exhibited the independent associations between HRV and poor nutritional status defined by serum albumin < 3.8 g/dL, total cholesterol < 100 mg/dL, body mass index < 23 kg/m^2^, bodyweight loss within six months > 10%, bodyweight loss within three months > 5%, and normalized protein catabolic rate < 1.1 g/kg BW/day. The current study disclosed ANS impairment in hemodialysis patients with poor nutritional status. The impaired ANS function might be a potential mechanism linking malnutrition to subsequent adverse prognoses in hemodialysis patients. Further investigations are warranted to confirm these findings and clarify the causal association among this complex issue.

## 1. Introduction

Nutritional assessment in patients with chronic kidney disease (CKD) is complex and requires a multidimensional workup including the patient’s dietary intake, bodyweight changes, comorbid conditions, and some relevant laboratory markers such as serum albumin, prealbumin, total cholesterol (T-Chol), and urine protein that can influence the nutritional status [1].

Protein-energy wasting (PEW) syndrome is a collective entity associated with higher morbidity and mortality in CKD patients. It is a state of progressively decreasing body stores of protein and energy fuels caused by several pathological processes and subsequent adaptative changes due to renal dysfunction [2]. To standardize the diagnosis of PEW syndrome, the International Society for Renal Nutrition and Metabolism (ISRNM) proposed diagnostic categories and criteria for PEW syndrome [2,3]. The incidence of PEW syndrome increases with worsening renal function, which is reported as 2.2%, 4.4%, 8.3%, 6.2%, 15.6%, and 24.6% in patients with CKD stage 1, 2, 3a, 3b, 4, and 5 without dialysis, respectively [4]. The incidence rate of PEW syndrome is even higher in patients with advanced CKD receiving maintenance dialysis (28–60%) [5] because of the additional protein losses elicited by dialysis [1,6]. The existence of PEW syndrome is associated with decreased quality of life, more morbidity, including adverse cardiovascular events, and increased all-cause mortality [7,8,9]. Thus, early recognition of CKD patients with malnutrition and appropriate interventions to improve their nutritional status is paramount in the aspect of patient care [1,2].

Heart rate variability (HRV) which reflects how the ANS responds to stimuli, is a simple, non-invasive method of assessing the autonomic nervous system (ANS). The higher HRV value denotes the better adaptive capacity and health [10]. HRV is measured by electrocardiographic recordings, which are analyzed to obtain indices in the time and frequency domains. Total power (TP) is the sum of the frequencies and represents the total autonomic output. Low-frequency (LF) or normalized LF (LF%) represents the sympathetic nervous activity. High-frequency (HF) or normalized HF (HF%) represents the parasympathetic one. The LF/HF ratio represents the sympathovagal balance or sympathetic nervous activity. Very-low-frequency (VLF) reflects the thermoregulation of the vasomotor tone. The variance of the R-R interval values (Var) represents all the components responsible for variability and reflects parasympathetic tone [11,12,13]. Alteration of HRV is associated with obesity [14], metabolic syndrome [15], and chronic diseases, including liver cirrhosis [10,16]. Abnormal ANS function is observed in nearly 81% of patients with advanced liver disease [16,17]. Decreased HRV in cirrhotic patients links to the severity of hepatic impairment, the degree of hepatic encephalopathy, and malnutrition, and subsequently contributes to significant mortality risks [16,17].

In CKD patients undergoing chronic hemodialysis, reduction of HRV is associated with higher morbidities, complications including vascular access failure and intradialytic hypotension, as well as higher mortality [18,19,20,21,22,23]. Nevertheless, the associations between HRV indices and nutritional markers have yet been adequately addressed in patients with CKD undergoing chronic hemodialysis. This study aimed to test the hypothesis that malnutrition is associated with altered ANS function, which might be one potential mechanism linking malnutrition to subsequent adverse prognoses in the CKD patients undergoing chronic hemodialysis.

## 2. Materials and Methods

### 2.1. Study Design and Population

This cross-sectional study was conducted using a cohort built in a teaching hospital in Northern Taiwan in 2010. The Institutional Review Board of Saint Mary Hospital Luodong approved this study (SMHIRB_105009). The study design conformed to the ethical guidelines of the 1975 Declaration of Helsinki. All the participants provided written informed consent, and all the data were analyzed anonymously.

Patients were included if they were adults who had been stable undergoing maintenance hemodialysis during the period from June to August 2010. Those who were less than 18 years old, had received hemodialysis less than three months from initiation, had an arrhythmia or active infections, or refused to receive HRV measurement were excluded.

All enrolled patients received HRV measurement before hemodialysis (HRV-0) and three times during hemodialysis (HRV-1, -2, and -3 at the initial, middle, and late phases of the index hemodialysis session, respectively). These participants were categorized into two groups according to the individual nutritional markers and their cut-off points. Then, we compared the differences of HRV indices between the two groups.

Baseline demographic data, comorbid diseases, causes of uremia, and medications were extracted from the patients’ medical records. Relevant clinical parameters were recorded, which include the cardiothoracic ratios from chest X-ray, laboratory examinations such as complete blood cell count, blood urea nitrogen, serum creatinine, electrolytes, albumin, glucose, glycated hemoglobin (HbA1c), lipid profiles, and intact-parathyroid hormone, and the efficiency parameters of hemodialysis such as Kt/V and urea reduction ratio. Diabetes mellitus was documented if the patients have been receiving oral hypoglycemic agents or insulin injections, or if their HbA1c was equal to or greater than 6.5% before treatment. The diagnosis of hypertension was established if the patients have been taking antihypertensive medications or if the measurements of their pre-dialytic blood pressure were higher than 140/90 mmHg over half of the time during the recent month [24]. These data mentioned above were gathered and documented at the time of HRV measurement.

### 2.2. Measurements of Heart Rate Variability

In the current study, values of HRV indices were obtained using a 5 min measurement of an HRV analyzer (SSIC, Enjoy Research Inc., Taipei, Taiwan) which has been applied in many published studies [15,19,20,25]. After keeping quiet for more than 20 min, patients received the HRV measurement when they lay quietly and breathed normally for 5 min. The analyzer records the signals from the lead I of electrocardiogram by using an 8-bit analog-to-digital converter with a sampling rate of 512 Hz. All peaks of the digitized electrocardiogram signals were detected using a spike detection algorithm and were analyzed online. The computer algorithm subsequently identified each QRS complex and excluded ventricular premature complexes and noise. Normal and stationary R-R interval values were resampled and interpolated at a rate of 7.11 Hz to produce continuity in the time domain, and a nonparametric method of fast Fourier transformation was used for power spectral analysis. After deleting the direct current component, a Hamming window was used to attenuate any leakage effects. The algorithm estimated the power density of the spectral components based on fast Fourier transformation. The resulting power spectrum was corrected for attenuation resulting from sampling and the application of the Hamming window. Then, the fast Fourier transformation was used to perform power spectral analysis, which quantified power spectrum into the standard frequency-domain measurements including VLF (0.003–0.04 Hz), LF (0.04–0.15 Hz), HF (0.15–0.40 Hz), TP, LF/HF ratio, and Var. The power spectrum of these HRV indices was logarithmically transformed for correcting the skewed distributions [18,25].

### 2.3. Nutritional Markers and Cut-Off Points

The nutritional markers and their cut-off values proposed in the diagnostic categories and criteria for the PEW syndrome by ISRNM (Table S1) [2,3] were selected in the current study.

These markers with respective cut-off values included serum albumin < 3.8 g/dL, total cholesterol < 100 mg/dL, body mass index (BMI) < 23 kg/m^2^, bodyweight loss within six months (BWL (6 m)) > 10%, bodyweight loss within three months (BWL (3 m)) > 5%, and normalized protein catabolic rate (nPCR) < 0.8 g/kg BW/day. Moreover, we additionally took nPCR < 1.1 g/kg BW/day, the median value of nPCR in our participants as a cut-off point for categorizing our participants.

### 2.4. Statistical Analyses

Statistical analyses were performed using the Scientific Package for Social Science (PASW Statistics for Windows, Version 18.0, SPSS Inc., Chicago, IL, USA). Independent Student’s *t*-tests were done to compare the differences in HRV indices between the two groups categorized by the cut-off points of all the proposed nutritional markers. Paired Student’s *t*-tests were used to compare the differences in HRV indices between different phases of hemodialysis in the same group.

The multivariate generalized estimating equation (GEE) was applied to determine the association between individual nutritional markers and individual HRV indices and was presented as an adjusted odds ratio (aOR) and 95% confidence interval (CI). We performed the statistical analyses by putting the individual HRV index one at a time, and many relevant clinical variables for adjustment, in the multivariate GEE model. Relevant variables were put in the multivariate model for adjustment. These factors included time variation of individual HRV indices (four HRV measurements), the nutritional markers, the baseline demographic data, the comorbid diseases, the causes of uremia, the medications, as well as laboratory data and clinical information at the index hemodialysis. Microsoft Office Excel 2013 was used to draw the plots showing the series of values of individual HRV indices of the two groups. Continuous data were expressed as mean ± standard deviation, whereas categorical variables were shown as number (percentage) unless otherwise specified. In all statistical analyses, a two-sided *p* ≤ 0.05 was considered statistically significant.

## 3. Results

During the period from 1 June to 31 August of 2010, a total of 202 patients who had been on maintenance hemodialysis for more than three months were screened. A total of 175 patients (100 women, mean age 65.1 ± 12.9 years) were enrolled. The baseline characteristics, comorbid conditions, etiology of uremia, and laboratory data of the enrolled patients were shown in Table 1.

### The Association between HRV Indices and Individual Nutritional Markers

Table 2 compared the values of HRV indices at four time points between the two groups, which were categorized by individual cut-off points of these nutritional markers.

Compared to the patients with serum albumin ≥ 3.8 g/dL, the patients with serum albumin < 3.8 g/dL had significantly lower values in some HRV indices at the baseline (VLF, LF%, and LF/HF ratio of HRV-0) and during the hemodialysis process (VLF and TP of HRV-1; and LF% of HRV-3). Compared to the patients with BMI ≥ 23 kg/m^2^, the patients with BMI < 23 kg/m^2^ had a significantly lower value of HF (of HRV-0) and HF% (of HRV-2 and HRV-3), but had higher values of VLF (of HRV-1), LF% and LF/HF ratio (of HRV-1, HRV-2, and HRV-3) during hemodialysis. Compared to the patients with nPCR ≥ 1.1 g/kg BW/day, those with nPCR < 1.1 g/kg BW/day had a significantly lower value of some HRV indices at the baseline (LF, TP, and Var of HRV-0), and had lower values in LF% (of HRV-1, HRV-2, and HRV-3) and LF/HF ratio (of HRV-3) but higher values in HF% (HRV-3). As to the comparisons of the two groups categorized by T-Chol of 100 mg/dL, BWL (6 m) of 10%, BWL (3 m) of 5%, and nPCR of 0.8 g/kg BW/day, there were no significant differences of HRV indices from HRV-0 to HRV-3.

**Figure 1:**
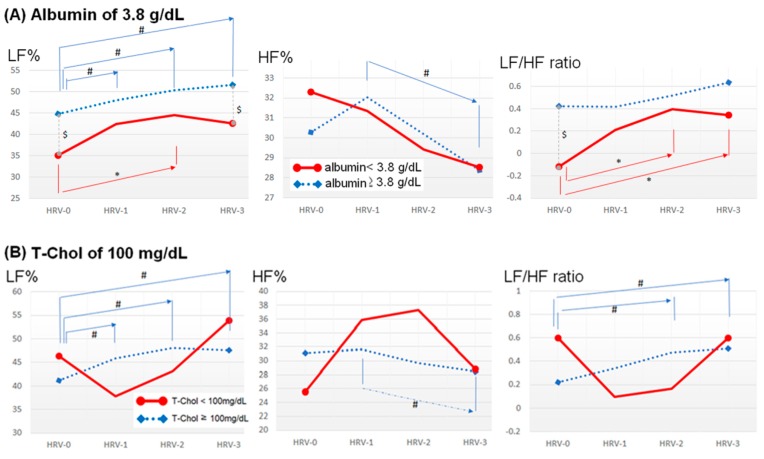

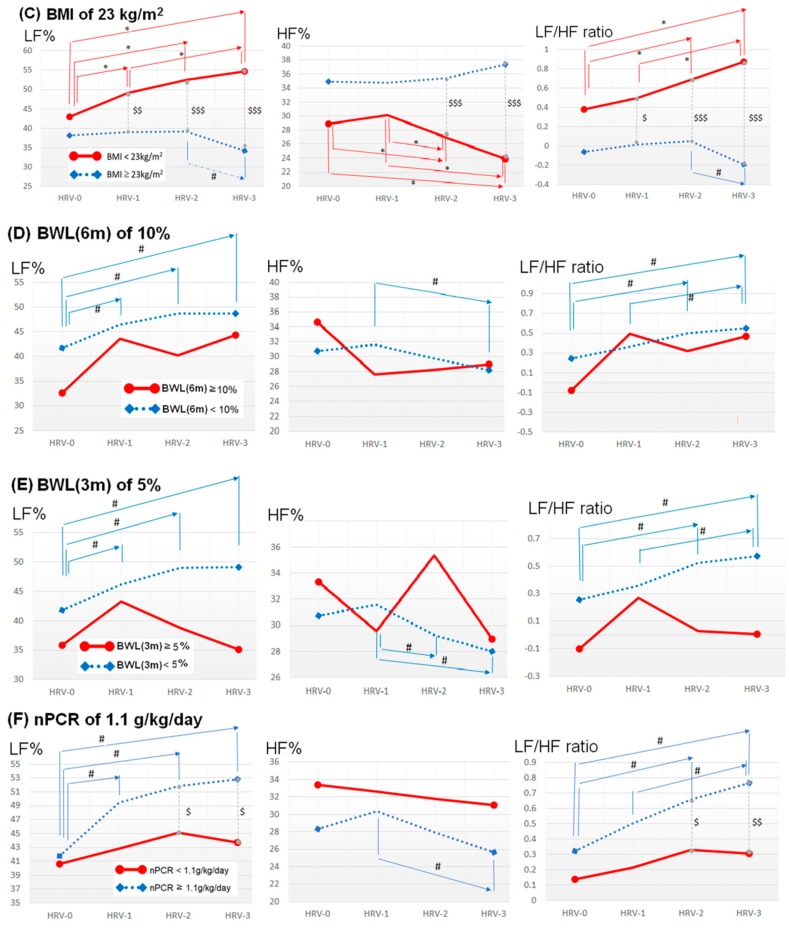
Serial changes of LF%, HF%, and LF/HF ratio of the two groups categorized by (**A**) serum albumin of 3.8 g/dL, (**B**) T-Chol of 100 mg/dL, (**C**) BMI of 23 kg/m^2^ (**D**), BWL (6m) of 10%, (**E**) BWL (3m) of 5%, and (**F**) nPCR of 1.1 g/kg BW/day. Independent and paired Student’s t-test were used to compare the differences of HRV values between the two groups and between different phases of hemodialysis in the same group, respectively. The solid red line and dotted blue line indicate the serial changes of the individual HRV index (LF%, HF%, and LF/HF ratio) in patients with worse and better nutritional states, respectively. $, $$, and $$$ denote *p* < 0.05, 0.01, and 0.001, respectively, in the comparisons of HRV values at the same time points between two groups. * and # denote *p* < 0.05 in the comparisons of HRV values at different time points of the group with the worse and better nutritional state, respectively. Abbreviations: BMI: Body mass index; BWL (6 m): Bodyweight loss within six months; BWL (3 m): Bodyweight loss within three months; HF%: Normalized high frequency; LF%: Normalized low frequency; LF/HF ratio: Low- frequency/high-frequency ratio; nPCR: Normalized protein catabolic rate; T-Chol: Total cholesterol.

Figure 1 illustrated the series changes of LF%, HF%, and LF/HF ratio during hemodialysis of two groups categorized by the individual nutritional markers. Regarding the trend of HRV values during the hemodialysis, the progressively increased values of LF% and/or LF/HF ratio were noticed in the patients with better nutritional status defined by albumin ≥ 3.8 g/dL, T-Chol ≥ 100 mg/dL, BWL (6 m) < 10%, BWL (3 m) < 5%, and nPCR ≥ 1.1 g/kg BW/day, as well as in those with worse nutritional status defined by BMI < 23 kg/m^2^. As to the comparisons between groups, the patients with serum albumin < 3.8 g/dL and nPCR < 1.1 g/kg BW/day had statistically lower values of LF% and LF/HF ratio than those with better nutritional status. Oppositely, the patients with BMI < 23 kg/m^2^ had statistically higher values of LF% and LF/HF ratio, along with a lower HF% value than those with BMI ≥ 23 kg/m^2^.

Subsequently, we used multivariate GEE to determine the independent association between nutritional markers and HRV indices. The relevant variables put in the multivariate model included the time variation of individual HRV indices (four HRV measurements), baseline demographic data (age, gender), comorbid diseases (diabetes mellitus, hypertension, coronary artery disease, heart failure, cerebrovascular disease, peripheral arterial disease), causes of uremia, medications (taking beta-blockers or angiotensin-converting enzyme inhibitor/angiotensin receptor blocker), laboratory data (blood urea nitrogen, creatinine, Kt/V, calcium, phosphate, sodium, potassium, intact parathyroid hormone, hemoglobin, white blood cell count, triglyceride, low-density lipoprotein, high-density lipoprotein, non-fasting sugar, glycated hemoglobin), clinical information at the index hemodialysis (cardiothoracic ratio, ultrafiltration divided by bodyweight (%UF), blood pressure, and hypotension event), as well as nutritional markers (albumin, T-Chol, BMI, BWL (6 m), BWL (3 m), nPCR).

All the HRV indices which were independently associated with worse nutritional status were listed in Table 3. “Serum albumin < 3.8 g/dL” was independently associated with lower VLF (aOR = 0.32, *p* = 0.003), TP (aOR = 0.32, *p* = 0.015), Var (aOR = 0.37, *p* = 0.028), and LF% (aOR = 6.34 × 10^−5^, *p* = 0.013). “T-Chol < 100 mg/dL” and “BMI < 23 kg/m^2^” were both associated with higher LF% (aOR= 3.35 x 10^20^ and 3.42 x 10^6^, respectively) and LF/HF ratio (aOR = 9.17 and aOR = 2.34, respectively), as well as lower HF% (aOR = 4.71 × 10^−7^,(*p* = 0.034) and 4.14 × 10^−6^, respectively). “BWL (6 m) ≥ 10%” and “BWL (3 m) ≥ 5%,” completely opposite to “T-Chol < 100 mg/dL” and “BMI < 23 kg/m^2^,” were both associated with lower LF% (aOR = 4.58 × 10^−17^ and 4.78 × 10^−17^, respectively) and LF/HF ratio (aOR = 0.17 and 0.13, respectively), as well as higher HF% (aOR = 7.38 × 10^5^ and 1.53 × 10^8^, respectively). Lastly, “nPCR < 1.1 g/kg BW/day” was independently associated with lower HF% (aOR = 0.01, *p* = 0.047). (All *p* < 0.001 unless otherwise denoted) (Table 3)

## 4. Discussion

To the best of our knowledge, this is the first study evaluating the independent association between nutritional status and ANS function in uremic patients undergoing chronic hemodialysis. Moreover, the current study calculated the effects of the four repeated HRV measurements, which added much more value in the statistical results since HRV tends to change under stress. We found that poor nutritional status defined by the six proposed nutritional markers exhibited the independent associations with different HRV values. These results indicated that these nutritional markers are clinically relevant in predicting ANS function in terms of the HRV.

### 4.1. Impact of Uremia on ANS

ANS moderates the adaptive changes to stimuli in order to maintain good health [10]. The ANS becomes progressively impaired in a patient with CKD as glomerular filtration rate declines, and impairment of ANS was found in more than 50% of the patients with advanced CKD [26,27,28,29]. The ANS abnormality is characterized by an overall reduction in HRV, overactivity in the sympathetic tone (LF% and LF/HF ratio) and reduction in parasympathetic tone (HF%) [26,27,28]. Moreover, the reduction in compensatory responses to stress is also observed. During stress, an initial sympathetic activation with increased general HRV would be followed by a sympathetic withdrawal toward the end or when the stress becomes intense or prolonged [27,30]. During hemodialysis, an adequate compensatory increase, especially in the sympathetic tone, indicates a better autonomic reserve and better survival [31]. On the other hand, an inadequate response predisposes patients to morbidities such as intradialytic hypotension and vascular access failure, or even mortality [18,19].

### 4.2. Impact of Nutritional Status on ANS

The “reverse epidemiology” phenomenon is observed in patients undergoing chronic hemodialysis, in which traditional risk factors for cardiovascular diseases such as high BMI, hypercholesterolemia are associated with lower mortality risk [32]. Notably, T-chol < 100 mg/dL and BMI < 23 kg/m^2^ were independent associated with increased values of sympathetic indices (LF% and LF/HF ratio) in the current study. Additionally, these associations were opposite to other nutritional markers (albumin < 3.8 g/dL, BWL (6 m) > 10%, BWL (3 m) > 5%, and nPCR < 1.1 g/kg BW/days) in which values of HRV indices (especially LF% and LF/HF) were generally decreased. Actually, the characteristic of ANS dysfunction is diverse, which includes either overall reduced HRV, or overactive sympathetic tone with reduced parasympathetic tone at initial, following a subsequent sympathetic withdrawal when facing stress [26,27,28,30]. Although the detailed mechanism linking “reverse epidemiology” and ANS is not yet clear, these findings may provide some inspiration in the issue.

In the current study, the compensatory increase of LF% to the stress during hemodialysis in patients with albumin ≥ 3.8 g/dL disappeared in those with serum albumin < 3.8 g/dL, suggesting that the impairment of autonomic reserve associates with lower serum albumin (Figure 1). Finally, the multivariate analysis proved that serum albumin < 3.8 g/dL was independently associated with both lower total ANS activity (TP and Var) and lower sympathetic activity (LF%) (Table 3). In uremic patients undergoing chronic hemodialysis, inflammation status, and inadequate nutrient intake are two major causes of low serum albumin [33,34]. Further detailed mechanisms include chronic metabolic acidosis, decreased synthesis, and increased catabolism of protein, increased vascular permeability, and inadequate nutrient intake. All of these, along with protein loss during dialysis, are responsible for the low serum albumin [22,35,36,37].

Similar to low serum albumin, both BWL (6 m) ≥ 10% and BWL (3 m) ≥ 5% were also found to have a significant association with impaired autonomic reserve (in LF% and LF/HF ratio), as well as an independent association with lower sympathetic activity (LF% and LF/HF ratio) and higher parasympathetic activity (HF%) (Figure 1 and Table 3). In the general population, obesity was associated with reduced HRV and increased cardiovascular risk, which can be reversed with weight reduction [14]. On the other hand, reduced HRV was also observed in underweight individuals [38]. In patients with chronic hemodialysis, the phenomenon termed “obesity paradox” also exists [39,40]. An increase of bodyweight and lean body mass is associated with better survival, while bodyweight loss links to increased mortality in hemodialysis patients [39,40,41]. Probably it is because the deleterious effect of malnutrition offsets the supposed risks of higher bodyweight to a certain degree until the cumulative cardiovascular risks exceed its protective effect [42]. The HRV changes of the current study provide a potential mechanism in ANS in support of the “obesity paradox.”

Interestingly, patients with BMI < 23 kg/m^2^ were disclosed to have a better autonomic reserve during the hemodialysis than those with a higher level (Figure 1). Additionally, BMI < 23 kg/m^2^ was independently associated with higher LF% and LF/HF ratio, and lower HF% (Table 3). Three potential explanations for these findings were proposed below. First, hemodialysis patients with BMI ≥ 23 kg/m^2^ are more likely to have central obesity, which is an entity that corresponded with lower sympathetic tone (LF% and LF/HF ratio) and higher parasympathetic activity (HF%) [43]. Second, a BMI ≥ 23 kg/m^2^ is associated with more signs of inflammation [44], and chronic inflammation leads to a general reduction in HRV indices [10,45,46] such as lower LF% and LF/HF ratio in our study. Third, fluid overload is an important issue in patients undergoing chronic hemodialysis. The amount of fluid present in these patients depends on their residual renal function, cardiac function, comorbidities, as well as their dietary habit and compliance to hemodialysis therapy, which are sometimes difficult to control. Fluid overload is especially prevalent in the population with DM and HF. In the current cohort, compared to patients with BMI < 23 kg/m^2^, patients with BMI ≥ 23 kg/m^2^ have significantly higher proportions of diabetes mellitus (48.3% versus 31.1%, *p* = 0.042) and non-significantly higher proportion of heart failure (31.0% versus 20.8%, *p* = 0.183). Since these two comorbidities are prone to fluid accumulation and loss of lean body mass, it is reasonable that the patients with higher BMI have higher hydration state. Ferrario et al. [47] demonstrated that the higher fluid status has a significant correlation with lower LF% and LF/HF ratio, as well as higher HF%. Fourth, the higher proportion of diabetes mellitus in patients with BMI ≥ 23 kg/m^2^ is responsible for the significant reduction in HRV in these patients. Although our findings had been adjusted to diabetes mellitus, heart failure, and cardiothoracic ratio, these explanations could at least partially interpret our findings that lower BMI (indicating lower fluid hydration status) were independently associated with higher LF% and LF/HF ratio, and lower HF%.

Hyperlipidemia is a well-known entity associated with the development and progression of autonomic neuropathy [48]. However, a paradoxical association between serum T-Chol level and patient survival exists [49]. Kilpatrick et al. [49] found that, in the cohort of 15,859 hemodialysis patients, both hyperlipidemia and hypolipidemia were associated with higher mortality risk, and the association became stronger among patients with hypoalbuminemia (<3.8 mg/dL patients and those with a lower nPCR (<1 g/kg BW/day). The proposed underlying mechanisms linking dyslipidemia and impaired HRV are oxidative stress and inflammatory mediators induced by hyperlipidemia [50]. Moreover, chronic inflammatory status results in a general reduction in HRV values [10,45,46]. These explanations are in line with our findings that patients with T-Chol < 100 mg/dL was independently associated with higher LF% and LF/HF ratio, and lower HF% than those with T-Chol ≥ 100 mg/dL (Table 3). Regarding the impact of lipid-lowering agents on the prognoses of hemodialysis patients, the 4D study did not disclose the survival benefit from lipid-lowering interventions in dialysis patients with diabetes [51], but conflicting results existed [52]. As to the influence of lipid-lowering agents on HRV, the use of statin drugs is associated with improvement in HRV (lower LF and higher HF) in health persons [53], whereas this influence persists in hemodialysis patients is unknown and warrants further investigation.

In addition to underlying processes contributing to the PEW syndrome, patients lose about 6–8 g of proteins per hemodialysis session, by inadvertent loss into the dialysate or by inflammatory changes elicited by hemodialysis [1,6], thus adequate protein intake becomes essential for them. In patients undergoing chronic hemodialysis, low protein intake was associated with increased risks of malnutrition and increased mortality [54,55]. In our study, statistical differences in HRV values were seen in the nutritional status categorized by nPCR < 1.1 g/kg BW/day but not with nPCR < 0.8 g/kg BW/day (Table 2). Moreover, the autonomic reserve (in LF% and LF/HF ratio) in patients with nPCR ≥ 1.1 g/kg BW/day disappeared in those with nPCR < 1.1 g/kg BW/day (Figure 1). These findings may suggest that in patients undergoing chronic hemodialysis, ANS impairment begins when patients’ nPCR below a higher level of 1.1 g/kg BW/day rather than a lower level of 0.8 g/kg BW/day. Since reduced autonomic reserve was associated with increased mortality [31], our findings at least partially provide a piece of the puzzle in explaining the elevated mortality in hemodialysis patients with lower protein intake.

**Figure 2:**
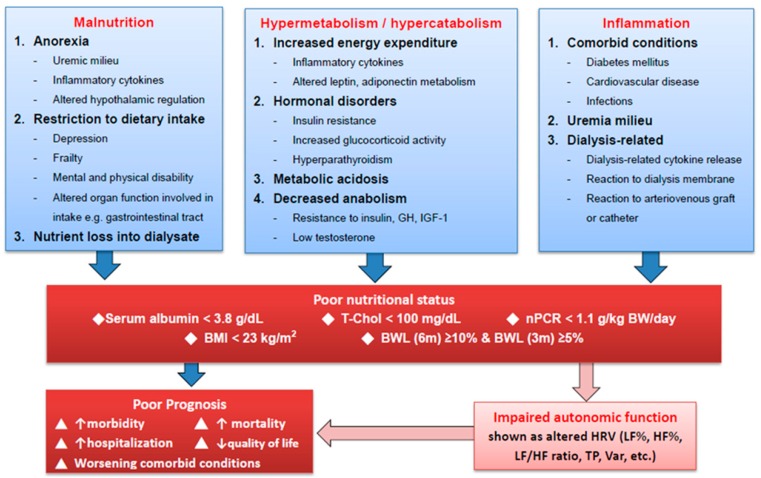
Proposed mechanism linking poor nutritional status to poor prognoses of hemodialysis patients. Abbreviations: BMI: Body mass index; BWL (6 m): Bodyweight loss within six months; BWL (3 m): Bodyweight loss within three months; GH: Growth hormone; HF%: Normalized high frequency; IGF-1: Insulin-like growth factor-1; LF%: Normalized low frequency; nPCR: Normalized protein catabolic rate; T-Chol: Total cholesterol; TP: Total power; Var: Variance of the R-R intervals.

Lastly, poor nutritional status results from the interplay of many factors presented in patients undergoing chronic hemodialysis and causes significant morbidity and mortality. Our study has demonstrated an association between poor nutritional status and impaired autonomic function, which further contributes negatively to the prognoses among these patients [18,19,31]. (Figure 2)

### 4.3. Limitations

Our study has some limitations. First, as a cross-sectional study, the current investigation aimed to explore the associations between nutritional markers and HRV values. Thus, the findings of the current study could not answer the questions regarding the direct causal relationship between these two entities, or the influence of drug intervention (i.e.,: T-Chol by statin) on HRV or patient prognoses. Second, the population of hemodialysis patients is heterogeneous in the patient nature. Several comorbid diseases such as diabetes mellitus and cardiovascular disease, as well as some medications such as beta-blockers and agents of the renin-angiotensin-aldosterone system blockade have influences on the HRV indices [15]. Nevertheless, we used multivariate GEE to demonstrate the independent association between individual nutritional markers and individual HRV indices by adjusting many variables, including baseline demographic data and comorbid diseases. The multivariate model excluded the confounding effects of these factors. Third, the patients in the current study represented a population with relatively good physical condition under adequate medical and dialysis care. The observations accrued here might not be extrapolated to uremic patients in worse conditions elsewhere. Fourth, although we took four HRV measurements into analysis for each patient, these HRV measurements were only performed during one index hemodialysis session and represented short-term changes, no data regarding long-term changes in HRV was available, and the bias of sampling could not be excluded. Fifth, fluid status and inflammation are two essential factors for consideration regarding nutritional markers [52]. In the current study, we took several variables (diabetes mellitus, heart failure cardiothoracic ratio, %UF, and blood pressure) to adjust the fluid status and white blood cell count to adjust inflammation status in the multivariate analysis. Some inflammatory markers such as c-reactive protein, or inflammatory cytokines such as interleukin-6 or tumor necrosis factor-α could provide relevant information about inflammation. Nevertheless, it is a shortcoming that these markers were not available in the current study.

## 5. Conclusions

The current study disclosed ANS impairment in hemodialysis patients with poor nutritional status defined by the proposed markers (serum albumin < 3.8 g/dL, T-Chol < 100 mg/dL, BMI < 23 kg/m^2^, BWL (6 m) ≥ 10%, BWL (3 m) ≥ 5% and nPCR < 1.1 g/kg BW/day). The impaired ANS function might be a potential mechanism linking malnutrition to subsequent adverse prognoses in patients undergoing hemodialysis. Further investigations are warranted to confirm these findings and clarify the causal association among this complex issue.

## Figures and Tables

**Table 1:** Characteristics of enrolled patients.

Total Enrolled Patients (*n* = 175)	
Age (years)	65.1 ± 12.9
Woman	100 (57.1%)
**Comorbidities** **and drugs**	
Diabetes mellitus	55 (31.4%)
Hypertension	128 (73.1%)
Taking beta-blockers or ACEi/ARB	56 (32.0%)
Heart failure	43 (24.6%)
Coronary artery disease	43 (24.6%)
Cerebrovascular disease	23 (13.1%)
Peripheral arterial disease	13 (7.4%)
**Causes of uremia**	
Diabetic nephropathy	55 (31.4%)
Hypertension	2 (1.1%)
Chronic glomerulonephritis	92 (52.6%)
Polycystic kidney disease	11 (6.3%)
Chronic interstitial nephritis	4 (2.3%)
Others	11 (6.3%)
**Baseline data**	
Cardio-thoracic ratio (%)	52.0 ± 5.0
Blood urea nitrogen (mg/dL)	74.7 ± 20.1
Creatinine (mg/dL)	10.5 ± 3.5
Kt/V	1.4 ± 0.2
Urea reduction ratio (%)	78.4 ± 54.8
Calcium (mg/dL)	9.1 ± 0.7
Phosphate (mg/dL)	4.9 ± 1.7
Calcium × Phosphate ((mg/dL)^2^)	44.6 ± 15.7
Sodium (mg/dL) Potassium (mEq/L)	137.8 ± 3.2 4.7 ± 0.8
Intact parathyroid hormone (ug/L)	285.5 ± 481.1
Hemoglobin (g/dL)	9.7 ± 1.4
White blood cell count (×10^9^/L)	6.3 ± 2.1
Triglyceride (mg/dL)	157.8 ± 132.0
Low-density lipoprotein (mg/dL)	98.1 ± 30.3
High-density lipoprotein (mg/dL)	35.6 ± 18.7
Sugar (non-fasting) (mg/dL)	148.2 ± 54.9
Glycated hemoglobin (%)	7.1 ±1.5
**Nutritional markers**	
Albumin (g/dL)	3.8 ± 0.3
Total cholesterol (mg/dL)	163.0 ± 35.5
Body mass index (kg/m^2^)	22.1 ± 3.9
BW loss within six months (kg)	0.0 ± 5.8
BW loss within three months (kg)	0.3 ± 4.7
nPCR (g/kg BW/day)	1.1 ± 0.4
**Data at the index hemodialysis**	
Dry weight (kg)	57.6 ± 29.6
Actual UF (kg)	2.22 ± 0.94
%UF (%)	4.02 ± 1.65
MAP at initial of hemodialysis	90.5 ± 17.0
sBP drop > 20 mmHg during hemodialysis	45 (25.7%)

Notes: Baseline laboratory data were the pre-dialysis data obtained before the patients receiving HRV measurement. Values are presented as mean ± standard deviation or number (%) unless otherwise stated. Abbreviations: ACEi: Angiotensin-converting enzyme inhibitor; ARB: Angiotensin receptor blocker; BMI: Body mass index; BWL (6 m): Bodyweight loss within six months; BWL (3 m): Bodyweight loss within three months; MAP: Mean arterial pressure; nPCR: Normalized protein catabolic rate; SBP: Systolic blood pressure; T-Chol: Total cholesterol; UF: Ultrafiltration; %UF: Ultrafiltration divided by bodyweight.

**Table 2:** Comparisons of the values of heart rate variability indices between the two groups categorized by nutritional markers.

	Serum chemistry		Body mass			Dietary intake	
	Albumin < 3.8 versus ≥ 3.8 g/dL	T-Chol < 100 versus ≥ 100 mg/dL	BMI < 23 versus ≥ 23 kg/m^2^	BWL (6 m) > 10% versus ≤ 10%	BWL (3 m) > 5% versus ≤ 5%	nPCR < 0.8 versus ≥ 0.8 g/kg BW/day	nPCR <1.1 versus ≥ 1.1 g/kg BW/day
**HRV-0**							
VLF	↓*	NS	NS	NS	NS	NS	NS
TP	NS	NS	NS	NS	NS	NS	↓*
Var	NS	NS	NS	NS	NS	NS	↓*
LF%	↓*	NS	NS	NS	NS	NS	NS
HF%	NS	NS	NS	NS	NS	NS	NS
LF/HF	↓*	NS	NS	NS	NS	NS	NS
**HRV-1**							
VLF	↓*	NS	↑*	NS	NS	NS	NS
TP	↓*	NS	NS	NS	NS	NS	NS
Var	NS	NS	NS	NS	NS	NS	NS
LF%	NS	NS	↑**	NS	NS	NS	↓*
HF%	NS	NS	NS	NS	NS	NS	NS
LF/HF	NS	NS	↑*	NS	NS	NS	NS
**HRV-2**							
VLF	NS	NS	NS	NS	NS	NS	NS
TP	NS	NS	NS	NS	NS	NS	NS
Var	NS	NS	NS	NS	NS	NS	NS
LF%	NS	NS	↑***	NS	NS	NS	↓*
HF%	NS	NS	↓***	NS	NS	NS	NS
LF/HF	NS	NS	↑***	NS	NS	NS	NS
**HRV-3**							
VLF	NS	NS	NS	NS	NS	NS	NS
TP	NS	NS	NS	NS	NS	NS	NS
Var	NS	NS	NS	NS	NS	NS	NS
LF%	↓*	NS	↑***	NS	NS	NS	↓*
HF%	NS	NS	↓***	NS	NS	NS	↑*
LF/HF	NS	NS	↑***	NS	NS	NS	↓*

Notes: HRV-0, -1, -2, and -3 were HRV measured before, and at the initial, middle, and late phases of the index hemodialysis session, respectively. The variables were compared after Log transformation. ↑ and ↓denote the higher and lower value of HRV indices in participants with worse nutritional status compared to those with better nutritional status. P-value was calculated using the independent Student’s *t*-test. *denotes *p* < 0.05, **denotes *p* < 0.01, ***denotes *p* < 0.001 in the comparison. Units: Ln (ms2) in VLF, LF, HF, TP, and Var; Ln (ratio) in LF/HF ratio; normalized units in LF% and HF%. Abbreviations: BMI: Body mass index; BWL (6 m): Bodyweight loss within six months; BWL (3 m): Bodyweight loss within three months; HF: High frequency; HRV: Heart rate variability; LF: Low frequency; HF%: Normalized high frequency; LF%: Normalized low frequency; nPCR: Normalized protein catabolic rate; NS: Not significant; T-Chol: Total cholesterol; TP: Total power; Var: Variance of the R-R intervals; VLF: Very low frequency.

**Table 3:** Heart rate variability indices independently associated with worse nutritional status.

**(A)**	**Serum albumin < 3.8 g/dL (Serum albumin ≥ 3.8 g/dL as reference)**				
	**HRV indices ^a^**	**B**	***p*-value**	**aOR**	**95% CI**
	VLF	−1.13	0.003	0.32	0.15–0.68
	TP	−1.15	0.015	0.32	0.12–0.80
	Var	−0.99	0.028	0.37	0.15–0.60
	LF%	−9.67	0.013	6.34 × 10^-5^	3.02 × 10^−8^–0.13
**(B)**	**T-Chol < 100 mg/dL (T-Chol ≥ 100 mg/dL as reference)**				
	**HRV indices ^a^**	**B**	***p*-value**	**aOR**	**95% CI**
	LF%	47.26	<0.001	3.35 × 10^20^	4.83 × 10^10^–2.32 × 10^30^
	HF%	−14.57	0.034	4.71 × 10^−7^	6.70 x 10^−13^–0.33
	LF/HF ratio	2.22	<0.001	9.17	2.80–30.05
**(C)**	**BMI < 23 kg/m^2^ (BMI ≥ 23 kg/m^2^ as reference)**				
	**HRV indices ^a^**	**B**	***p*-value**	**aOR**	**95% CI**
	HF	−1.03	0.031	0.36	0.14–0.91
	LF%	15.04	<0.001	3.42 × 10^6^	6.28 × 10^3^–1.86 × 10^9^
	HF%	−12.40	<0.001	4.14 × 10^−6^	1.27 × 10^−7^–1.35 × 10^−4^
	LF/HF ratio	0.86	<0.001	2.34	1.72–3.27
**(D)**	**BWL (6 m) ≥ 10% (BWL (6 m) < 10% as reference)**				
	**HRV indices ^a^**	**B**	***p*-value**	**aOR**	**95% CI**
	LF%	−37.62	<0.001	4.58 × 10^−17^	7.25 × 10^−22^–2.89 × 10^−12^
	HF%	13.51	<0.001	7.38 × 10^5^	9.21 × 10^2^–5.92 × 10^8^
	LF/HF ratio	−1.77	<0.001	0.17	0.09–0.32
**(E)**	**BWL (3 m) ≥ 5% (BWL (3 m) < 5% as reference)**				
	**HRV indices ^a^**	**B**	***p*-value**	**aOR**	**95% CI**
	LF%	−37.58	<0.001	4.78 × 10^−17^	3.50 × 10^−21^–6.51 × 10^−13^
	HF%	18.84	<0.001	1.53 × 10^8^	3.63 × 10^5^–6.42 × 10^10^
	LF/HF ratio	−2.07	<0.001	0.13	0.08–0.20
**(F)**	**nPCR < 1.1 g/kg BW/day (nPCR ≥ 1.1 g/kg BW/day as reference)**				
	**HRV indices ^a^**	**B**	***p*-value**	**aOR**	**95% CI**
	HF%	−5.21	0.047	0.01	3.23 × 10^5^–0.93

Note: The analyses were performed by putting time variation of individual HRV indices one at a time, and many relevant clinical variables for adjustment, in the multivariate generalized estimating equation. ^a^ every increment of one unit. Units: Ln (ratio) in LF/HF ratio; normalized units in LF% and HF%. Abbreviations: aOR: Adjusted odds ratio; BMI: Body mass index; BW: Body weight; BWL (6 m): Bodyweight loss within six months; BWL (3 m): Bodyweight loss within three months; CI: Confidence interval; HF%: Normalized high frequency; HRV: Heart rate variability; LF%: Normalized low frequency; LF/HF ratio: Low frequency/ high frequency ratio; nPCR: Normalized protein catabolic rate; T-chol: Total cholesterol.

## Supplementary Materials

The following are available online at https://www.mdpi.com/2077-0383/8/10/1700/s1. Table S1. Diagnostic categories and criteria for protein-energy wasting syndrome [3]. Note: International Society for Renal Nutrition and Metabolism (ISRNM) dictates that at least three out of the four categories and at least one in each category must be met for the diagnosis of protein-energy wasting syndrome. ^a^ Not valid if there are conditions or medications that can influence serum chemistry such as ongoing urinary or gastrointestinal protein losses or cholesterol-lowering agents.
