# Supplementary material for: The Association between Nutritional Markers and Heart Rate Variability Indices in Patients Undergoing Chronic Hemodialysis
Source: J Clin Med. 2019 Oct 16;8(10):1700. doi: 10.3390/jcm8101700 (PMC6832240; doi:10.3390/jcm8101700)
Supplement: Supplementary file 1 [file jcm-08-01700-s001.pdf]

Journal of
Clinical Medicine

Supplementary Information

Table 1. Diagnostic categories and criteria for protein-energy wasting syndrome [3].

Categories	Criteria within categories
Low serum chemistry	● Serum albumin < 3.8 g/dL (Bromcresol Green method) [a]<br>● Serum cholesterol < 100 mg/dL [a]<br>● Serum prealbumin < 30 mg/dL [a]
Low body mass	● Body mass index < 23 kg/m^2<br>● Unintentional weight loss over time: 10% over 6 months or 5% over 3 months<br>● Body fat percentage < 10%
Low muscle mass	● Muscle wasting: reduced muscle mass 5% over 3 months or 10% over 6 months<br>● Reduced mid-arm muscle circumference area (reduction > 10% in relation to 50th percentile of reference population, measured by a trained athropometrist<br>● Creatinine appearance
Low dietary intake	● Unintentional dietary protein intake < 0.60g/kg/day in chronic kidney disease stages 2–5 and <0.80g/kg/day in patient with maintenance hemodialysis for at least 2 months<br>● Unintentional dietary energy intake < 25kcal/kg/day for at least 2 months

Note: International Society for Renal Nutrition and Metabolism (ISRNM) dictates that at least three out of the four categories and at least one in each category must be met for the diagnosis of protein-energy wasting syndrome. [a] Not valid if there are conditions or medications that can influence serum chemistry such as ongoing urinary or gastrointestinal protein losses or cholesterol-lowering agents.

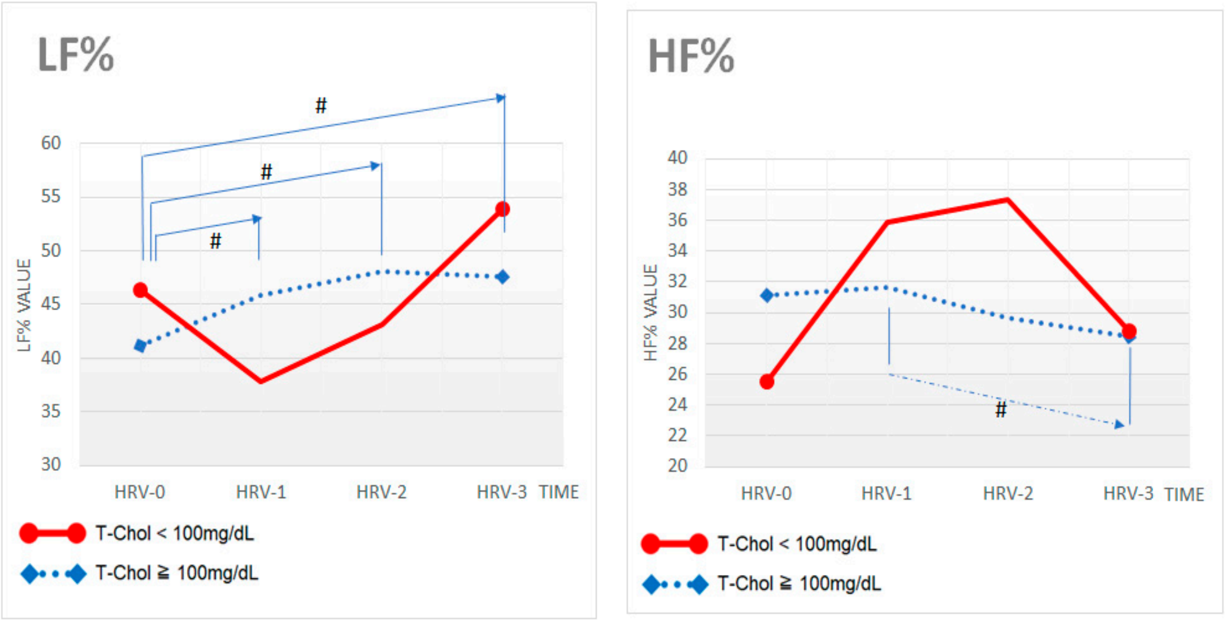

(**A**)

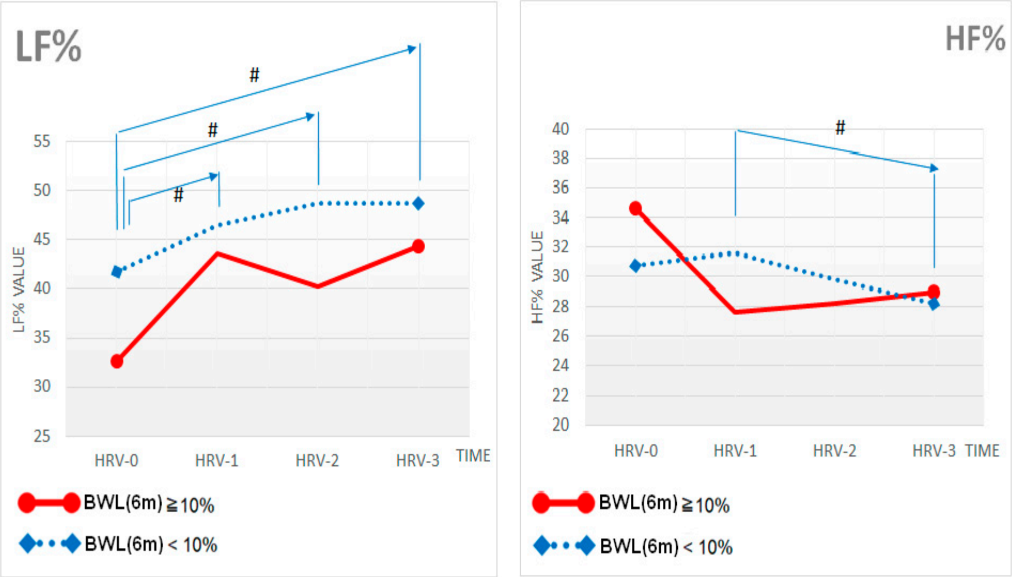
LF%
#
#
#
55
50
45
40
35
30
25
LF% VALUE
HRV-0
HRV-1
HRV-2
HRV-3
TIME
BWL(6m) ≧ 10%
BWL(6m) < 10%
HF%
#
40
38
36
34
32
30
28
26
24
22
20
HF% VALUE
HRV-0
HRV-1
HRV-2
HRV-3
TIME
BWL(6m) ≧ 10%
BWL(6m) < 10%

(**B**)

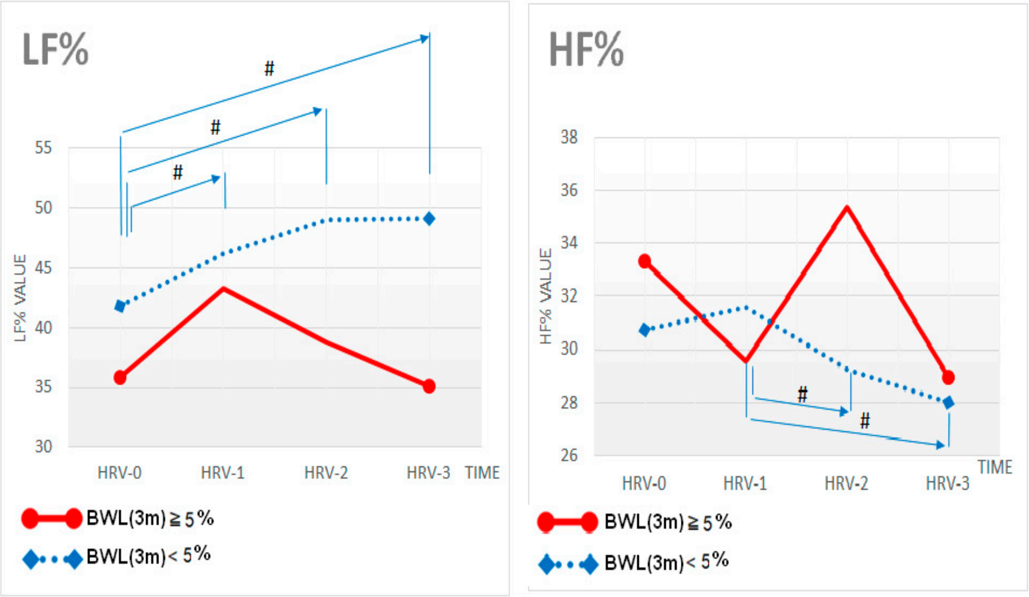
LF%
#
#
#
55
50
45
40
35
30
LF% VALUE
HRV-0
HRV-1
HRV-2
HRV-3
TIME
BWL(3m) ≧ 5%
BWL(3m) < 5%
HF%
38
36
34
32
30
28
26
HF% VALUE
#
#
HRV-0
HRV-1
HRV-2
HRV-3
TIME
BWL(3m) ≧ 5%
BWL(3m) < 5%

(**C**)

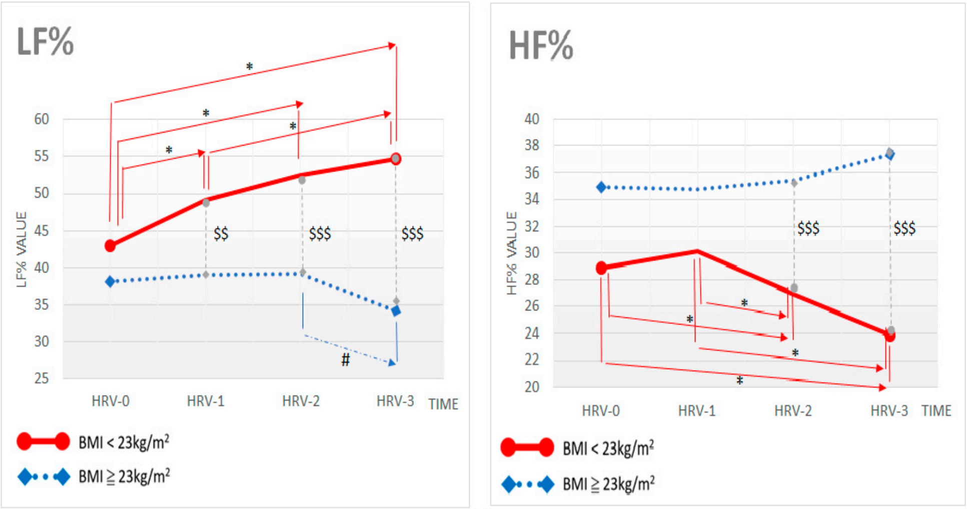

LF%
60
55
50
45
40
35
30
25
LF% VALUE
HRV-0
HRV-1
HRV-2
HRV-3
TIME
*
$$
$$$
#
BMI < 23kg/m²
BMI ≧ 23kg/m²
HF%
40
38
36
34
32
30
28
26
24
22
20
HF% VALUE
HRV-0
HRV-1
HRV-2
HRV-3
TIME
$$$
*
BMI < 23kg/m²
BMI ≧ 23kg/m²

(D)

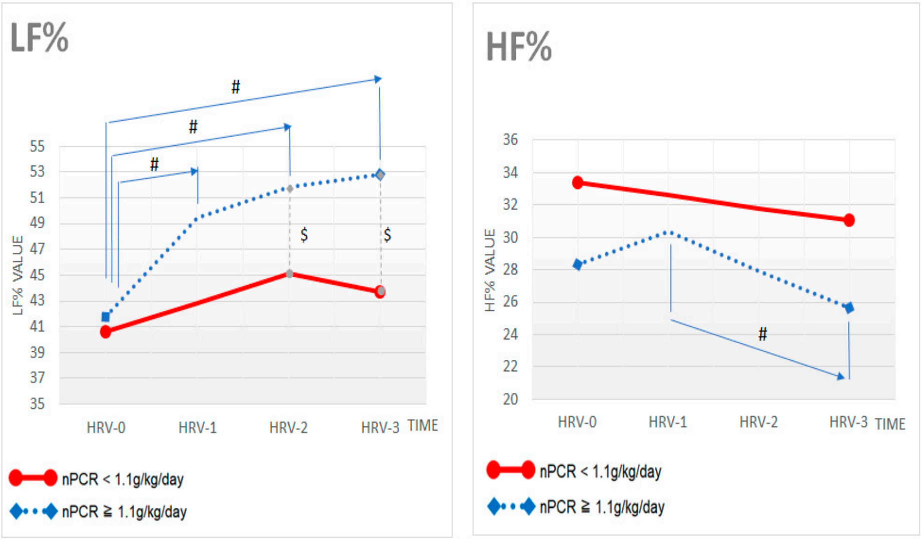

LF%
55
53
51
49
47
45
43
41
39
37
35
LF% VALUE
HRV-0
HRV-1
HRV-2
HRV-3
TIME
#
$
nPCR < 1.1g/kg/day
nPCR ≧ 1.1g/kg/day
HF%
36
34
32
30
28
26
24
22
20
HF% VALUE
HRV-0
HRV-1
HRV-2
HRV-3
TIME
#
nPCR < 1.1g/kg/day
nPCR ≧ 1.1g/kg/day

(E)

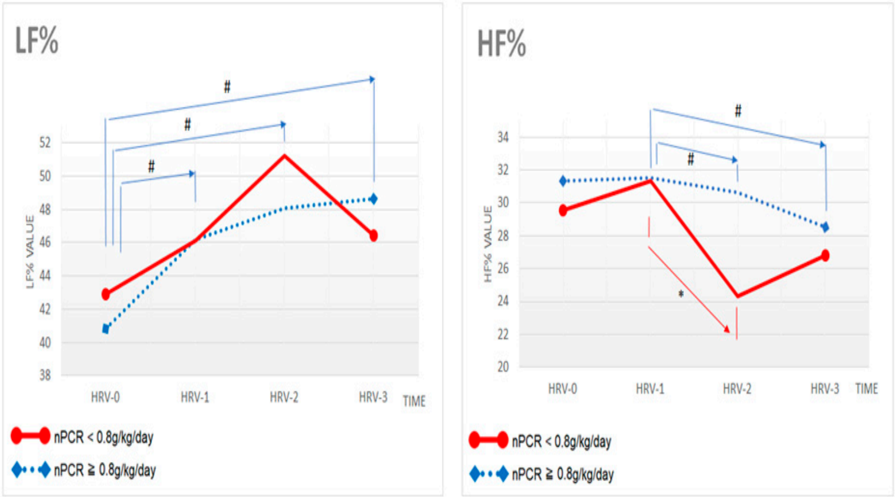

LF%
52
50
48
46
44
42
40
38
LF% VALUE
HRV-0
HRV-1
HRV-2
HRV-3
TIME
#
nPCR < 0.8g/kg/day
nPCR ≧ 0.8g/kg/day
HF%
34
32
30
28
26
24
22
20
HF% VALUE
HRV-0
HRV-1
HRV-2
HRV-3
TIME
#
*
nPCR < 0.8g/kg/day
nPCR ≧ 0.8g/kg/day

(F)

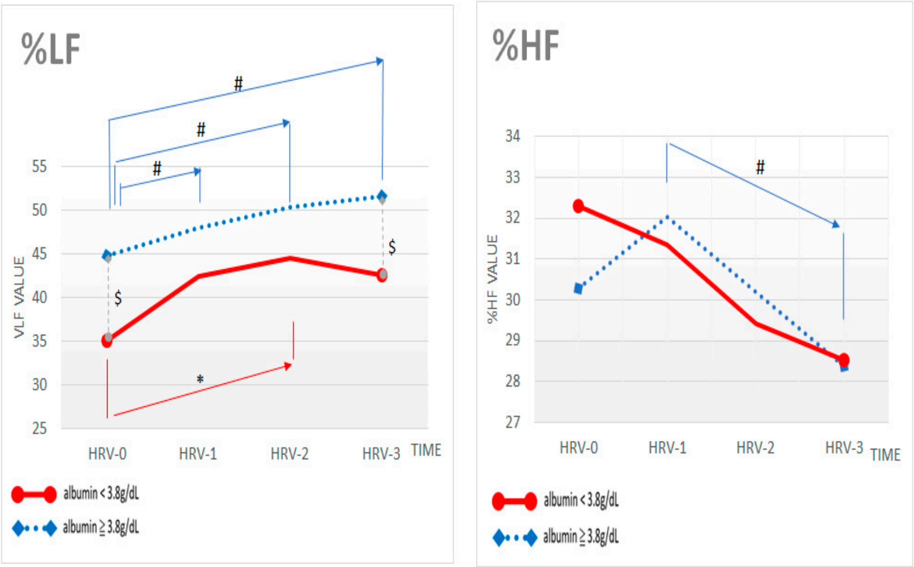

(**G**)

Figure S1. Serial changes of LF% and HF% two groups categorized by (**A**) T-Chol of 100 mg/dL, (**B**) BWL of 10% within 6 months, (**C**) BWL of 5% within 3 months, (**D**) BMI of 23 kg/m^2, (**E**) nPCR of 1.1 g/kg BW/day, (**F**) nPCR of 0.8 g/kg BW/day, and (**G**) serum albumin of 3.8 g/dL. Note: The analyses were done using independent and paired Student's *t*-test to compare the differences in HRV values between the two groups and between different phases of hemodialysis in the same group, respectively. The red solid line and blue dotted line indicate the serial changes of the specified HRV index in patients with worse and better nutritional states, respectively. $, $$ and $$$ denote $p < 0.05$, 0.01 and 0.001, respectively, in the comparisons of HRV values at the same time points between two groups. * denotes $p < 0.05$, in the comparisons of HRV values at different time points of the group with worse nutritional state. # denotes $p < 0.05$ in the comparisons of HRV values at different time points of the group with better nutritional state. Abbreviations: BMI, body mass index; BWL (6 m), body weight loss within 6 months; BWL (3 m), body weight loss within 3 months; HF%, normalized high frequency; LF%, normalized low frequency; nPCR, normalized protein catabolic rate; T-Chol, total cholesterol.

© 2019 by the authors. Licensee MDPI, Basel, Switzerland. This article is an open access article distributed under the terms and conditions of the Creative Commons Attribution (CC BY) license (http://creativecommons.org/licenses/by/4.0/).
